# Supplementary figures and images for: A preliminary assessment of population genetic structure of the common vampire bat (Desmodus rotundus) in Colombia
Source: PeerJ. 2025 Nov 10;13:e20306. doi: 10.7717/peerj.20306 (PMC12614099; doi:10.7717/peerj.20306)

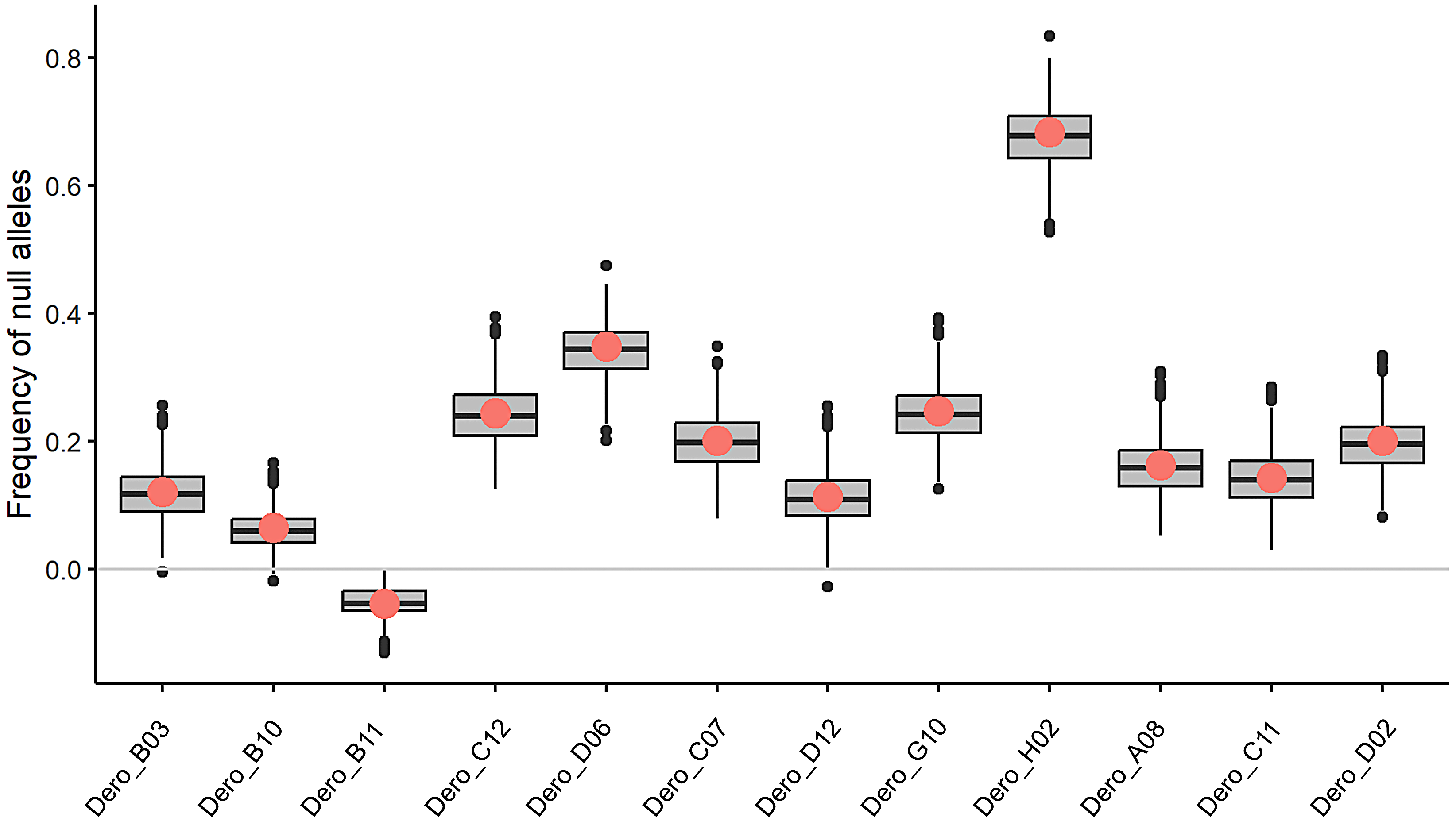

Supplement: Supplemental Information 1 — Estimated frequencies of null alleles at each of the 12 microsatellite loci for Desmodus rotundus. Null allele frequency was estimated using the Brookfield (1996) method in MicroChecker (Van Oosterhout et al., 2004) . Loci with null allele frequency >0.35 were eliminated from consideration (i.e., locus Dero_H02). [file peerj-13-20306-s001.png]

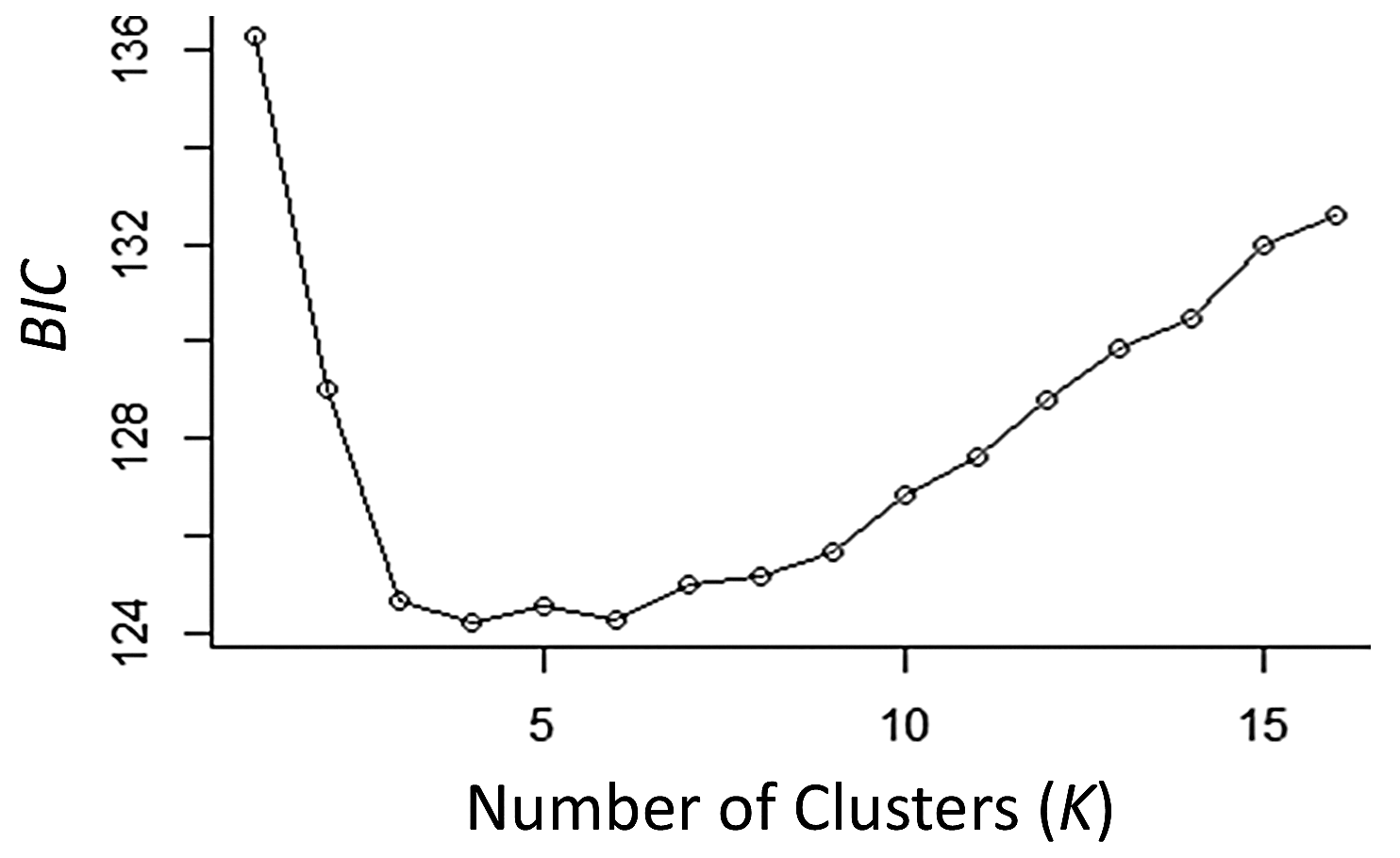

Supplement: Supplemental Information 2 — Bayesian information criterion (BIC) model selection criterion for progressive numbers of DAPC clusters (K) for identification of the best-supported number of clusters within our samples. We tested one to 15 clusters (three more than the number of sampling sites). Results indicate that three clusters of genetically similar individuals (i.e. populations) are most likely present within the data. [file peerj-13-20306-s002.png]

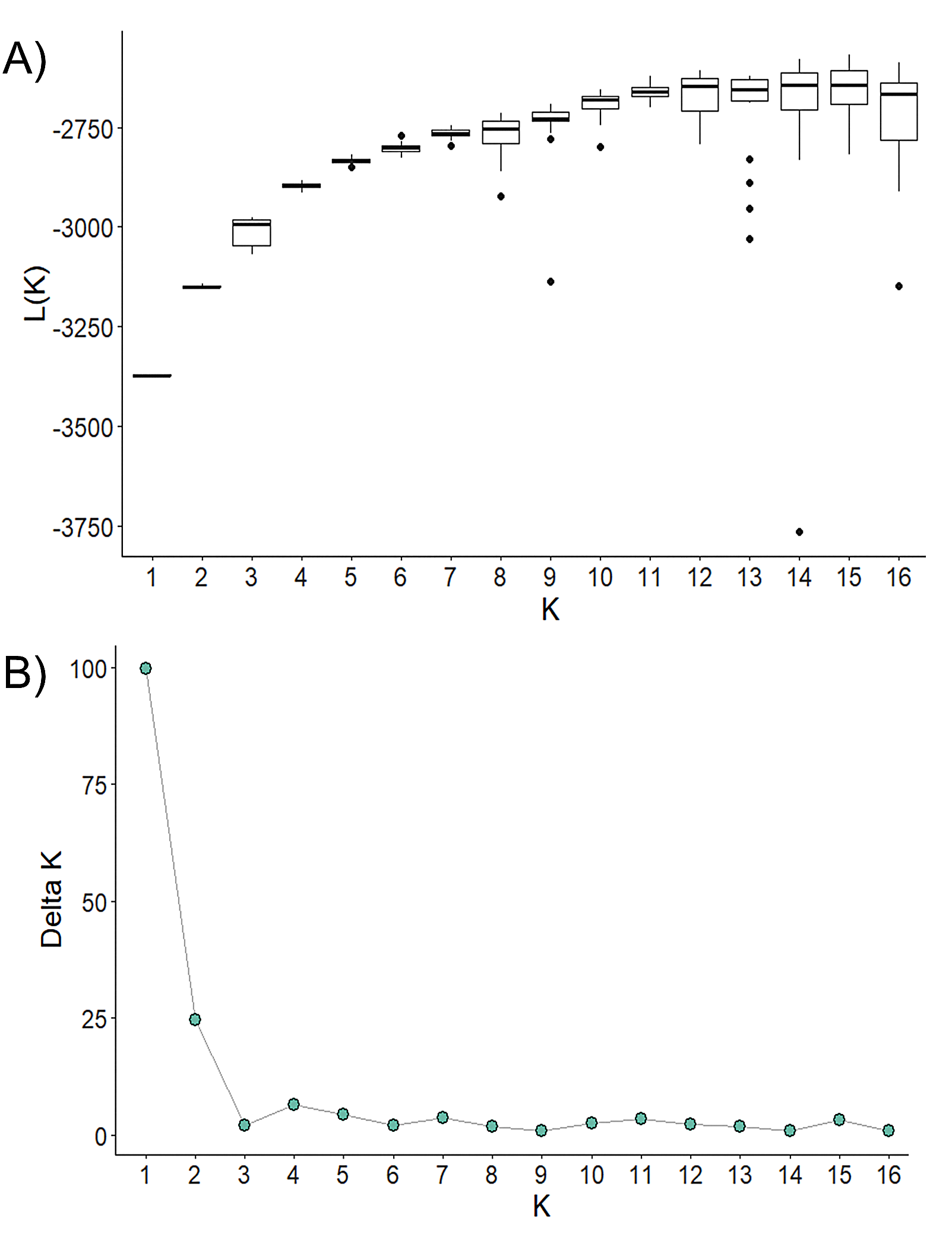

Supplement: Supplemental Information 3 — Analysis of STRUCTURE-based clusters (K) (Pritchard, Stephens & Donnelly, 2000) conducted using the delta K method of Evanno et al. (2005). A) Mean likelihood of each cluster from 20 replicate runs for each value of K. B) Delta K (mean absolute value of average likelihood over 20 runs divided by the standard deviation of mean likelihood) of each progressive number of clusters. Analysis of delta K was performed using STRUCTURE version 2.3.4 (Pritchard, Stephens & Donnelly, 2000). [file peerj-13-20306-s003.png]
