# Supplementary material for: A preliminary assessment of population genetic structure of the common vampire bat (Desmodus rotundus) in Colombia
Source: PeerJ. 2025 Nov 10;13:e20306. doi: 10.7717/peerj.20306 (PMC12614099; doi:10.7717/peerj.20306)
Supplement: Supplemental Information 4 — Sampling sites (i.e. locations of collection) were located across an elevational gradient from low (<500 meters in elevation), to moderate (500–1,000 meters in elevation) to high (>1,000 meters in elevation). This elevational gradient was used as a proxy for environmental variation through which we assessed multiple factors as they relate to the structure of population genetics of D. rotundus in Colombia after analysis. Samples for this study were collected in the summers of 2022 and 2023. Five samples were provided by the Instituto de Investigación de Recursos Biológicos Alexander von Humboldt (IAvH) (Bogotá, Colombia) and were collected in Colombia between 2019 and 2022. As a comparator, we amplified DNA from six D. rotundus individuals collected in western Mexico in 2007 by Piaggio, Johnson & Perkins (2008). [file peerj-13-20306-s004.docx]

**Table S1: Sample sites for collection of tissue from *Desmodus rotundus* individuals.** Sampling sites (i.e. locations of collection) were located across an elevational gradient from low (<500 meters in elevation), to moderate (500-1000 meters in elevation) to high (> 1000 meters in elevation). This elevational gradient was used as a proxy for environmental variation through which we assessed multiple factors as they relate to the structure of population genetics of *D. rotundus* in Colombia after analysis. Samples for this study were collected in the summers of 2022 and 2023. Five samples were provided by the Instituto de Investigación de Recursos Biológicos Alexander von Humboldt (IAvH) (Bogotá, Colombia) and were collected in Colombia between 2019 and 2022. As a comparator, we amplified DNA from six *D. rotundus* individuals collected in western Mexico in 2007 by Piaggio et al. (2008).

| **Sampling Site**  **(Department)** | **N** | **Source** | **Latitude (Decimal Degrees)** | **Longitude (Decimal Degrees)** | **Elevation (m)** |
| --- | --- | --- | --- | --- | --- |
| Agua de Dios (Cundinamarca) | 40 | This Study, Colombia | 4.35107 | -74.6516 | 407.80 |
| Los Araguatos  Centro Recreacional, (Arauca) | 1 | IAvH, Colombia | 6.41163 | -69.8551 | 127.00 |
| Yopal (Casanare) | 1 | IAvH, Colombia | 5.88064 | -71.8929 | 151.00 |
| Chaparral (Tolima) Site 1 | 6 | This Study, Colombia | 3.64442 | -74.4784 | 615.00 |
| Chaparral (Tolima) Site 2 | 3 | This Study, Colombia | 3.61964 | -75.49315 | 654.00 |
| Coello (Tolima) | 7 | This Study, Colombia | 4.24741 | -74.9761 | 439.20 |
| Vereda El Peñon, El Porvenir,  (Córdoba) | 1 | IAvH, Colombia | 9.374642 | -75.7619 | 36.16 |
| Ibagué (Tolima) | 5 | This Study, Colombia | 4.57583 | -75.3265 | 2198.90 |
| Medina (Cundinamarca) | 1 | This Study, Colombia | 4.5084 | -73.3499 | 760.00 |
| Nuevo Leon | 2 | A. Piaggio, Mexico | 25.32615 | -100.159 | 1200.00 |
| Piedras (Tolima) | 6 | This Study, Colombia | 4.44218 | -74.9877 | 619.10 |
| Pipiral (Meta) | 5 | This Study, Colombia | 4.204709 | -73.7138 | 965.30 |
| Puente Quetame (Cundinamarca) | 7 | This Study, Colombia | 4.30932 | -73.8788 | 1890.00 |
| Puerto Gaitán (Meta) | 1 | IAvH, Colombia | 4.312969 | -72.0836 | 207.00 |
| San Martín (Meta) | 2 | This Study, Colombia | 3.531883 | -73.4025 | 297.30 |
| Tamaulipas | 4 | A. Piaggio, Mexico | 23.96592 | -99.3659 | 623.00 |
