# Supplementary material for: A preliminary assessment of population genetic structure of the common vampire bat (Desmodus rotundus) in Colombia
Source: PeerJ. 2025 Nov 10;13:e20306. doi: 10.7717/peerj.20306 (PMC12614099; doi:10.7717/peerj.20306)
Supplement: Supplemental Information 5 — FST and ${G}_{ST}^{{^{\prime}}}$GST′ metrics of genetic distance between each sampling site of D. rotundus individuals. Greater genetic distance is signified by large numbers. Sampling sites Nuevo Leon and Tamaulipas were located in Mexico and were used as a comparator for all other sites located in Colombia. [file peerj-13-20306-s005.docx]

**Table S2. Metrics of genetic distance between sampling sites.** *F*_ST_ and *G*’_ST_ metrics of genetic distance between each sampling site of *D. rotundus* individuals. Greater genetic distance is signified by large numbers. Sampling sites Nuevo Leon and Tamaulipas were located in Mexico and were used as a comparator for all other sites located in Colombia.

| *G*’_ST_ | | | | | | | | | | | | | | | | |
| --- | --- | --- | --- | --- | --- | --- | --- | --- | --- | --- | --- | --- | --- | --- | --- | --- |
| *F*_ST_ |  | Agua de Dios | Arauca | Yopal | Chaparral | Coello | El Porvenir | Ibagué | Medina | Nuevo León | Piedras | Pipiral | Puente Quetame | Puerto Gaitán | San Martín | Tamaulipas |
|  | Agua de Dios (Cundinamarca) | - | 0.73 | 0.53 | 0.14 | 0.06 | 0.48 | 0.07 | 0.54 | 0.79 | 0.18 | 0.29 | 0.23 | 0.52 | 0.06 | 0.80 |
|  | Los Araguatos (Arauca) | 0.20 | - | 0.68 | 0.71 | 0.65 | 0.91 | 0.72 | 0.52 | 0.88 | 0.82 | 0.60 | 0.61 | 0.94 | 0.66 | 0.95 |
|  | Yopal (Casanare) | 0.11 | 0.00 | - | 0.61 | 0.51 | 0.88 | 0.46 | 0.68 | 0.88 | 0.72 | 0.41 | 0.53 | 0.71 | 0.42 | 0.93 |
|  | Chaparral (Tolima) | 0.04 | 0.21 | 0.17 | - | 0.14 | 0.52 | 0.18 | 0.61 | 0.84 | 0.34 | 0.35 | 0.36 | 0.62 | 0.12 | 0.82 |
|  | Coello (Tolima) | 0.00 | 0.13 | 0.03 | 0.02 | - | 0.56 | 0.04 | 0.61 | 0.77 | 0.34 | 0.27 | 0.19 | 0.63 | 0.18 | 0.83 |
|  | El Porvenir (Córdoba) | 0.09 | 0.00 | 0.00 | 0.13 | 0.07 | - | 0.62 | 0.81 | 0.88 | 0.60 | 0.59 | 0.74 | 0.70 | 0.59 | 0.91 |
|  | Ibagué (Tolima) | 0.00 | 0.16 | 0.00 | 0.04 | 0.00 | 0.10 | - | 0.62 | 0.69 | 0.29 | 0.32 | 0.21 | 0.64 | 0.07 | 0.76 |
|  | Medina (Cundinamarca) | 0.12 | 0.00 | 0.00 | 0.17 | 0.10 | 0.00 | 0.10 | - | 0.92 | 0.70 | 0.50 | 0.40 | 0.85 | 0.18 | 0.94 |
|  | Nuevo Leon | 0.23 | 0.16 | 0.18 | 0.26 | 0.18 | 0.16 | 0.17 | 0.18 | - | 0.83 | 0.72 | 0.77 | 0.98 | 0.82 | -0.02 |
|  | Piedras (Tolima) | 0.05 | 0.30 | 0.25 | 0.12 | 0.09 | 0.17 | 0.08 | 0.24 | 0.29 | - | 0.45 | 0.43 | 0.77 | 0.36 | 0.84 |
|  | Pipiral (Meta) | 0.08 | 0.04 | 0.00 | 0.09 | 0.01 | 0.00 | 0.04 | 0.00 | 0.09 | 0.13 | - | 0.19 | 0.48 | 0.18 | 0.77 |
|  | Puente Quetame (Cundinamarca) | 0.07 | 0.11 | 0.09 | 0.11 | 0.02 | 0.21 | 0.04 | 0.02 | 0.22 | 0.15 | 0.02 | - | 0.60 | 0.05 | 0.80 |
|  | Puerto Gaitan, Meta | 0.13 | 0.00 | 0.00 | 0.19 | 0.13 | 0.00 | 0.13 | 0.00 | 0.22 | 0.28 | 0.00 | 0.17 | - | 0.39 | 0.93 |
|  | San Martín (Meta) | 0.00 | 0.00 | 0.00 | 0.01 | 0.00 | 0.00 | 0.00 | 0.00 | 0.13 | 0.09 | 0.00 | 0.00 | 0.00 | - | 0.85 |
|  | Tamaulipas | 0.29 | 0.44 | 0.45 | 0.31 | 0.31 | 0.44 | 0.28 | 0.45 | 0.00 | 0.36 | 0.24 | 0.30 | 0.44 | 0.33 | - |
