# Supplementary material for: A preliminary assessment of population genetic structure of the common vampire bat (Desmodus rotundus) in Colombia
Source: PeerJ. 2025 Nov 10;13:e20306. doi: 10.7717/peerj.20306 (PMC12614099; doi:10.7717/peerj.20306)
Supplement: Supplemental Information 6 — Metrics of effective migration between collection sites in Colombia using both Wright’s FST equation for effective migration (m) (Wright, 1951; Zhivotovsky, 2015). Effective migration or “equilibrium” migration rates can be interpreted as individuals migrants per generation across ecological time calculated using FST, which is a summary statistic of genetic differentiation (Wang & Whitlock, 2023; Yamamichi & Innan, 2012). High migration rates (>1 individual per generation) was present between sites surrounding the Río Magdalena valley. [file peerj-13-20306-s006.docx]

**Table S3: Effective Migration Rate (*m*) between sampling sites.** Metrics of effective migration between collection sites in Colombia using both Wright’s *F_ST_* equation for effective migration (*m*) (Wright, 1951; Zhivotovsky, 2015). Effective migration or “equilibrium” migration rates can be interpreted as individuals migrants per generation across ecological time calculated using *F_ST_*, which is a summary statistic of genetic differentiation (Wang & Whitlock, 2003; Yamamichi & Innan, 2012). High migration rates (>1 individual per generation) was present between sites surrounding the Río Magdalena valley.

|  | Agua de Dios | Arauca | Yopal | Chaparral | Coello | El Porvenir | Ibagué | Medina | Nuevo León | Piedras | Pipiral | Puente Quetame | Puerto Gaitán | San Martín |
| --- | --- | --- | --- | --- | --- | --- | --- | --- | --- | --- | --- | --- | --- | --- |
| Los Araguatos (Arauca) | 0.02 | - |  |  |  |  |  |  |  |  |  |  |  |  |
| Yopal (Casanare) | 0.05 | 0.00 | - |  |  |  |  |  |  |  |  |  |  |  |
| Chaparral (Tolima) | 0.15 | 0.02 | 0.03 | - |  |  |  |  |  |  |  |  |  |  |
| Coello (Tolima) | 0.00 | 0.04 | 0.18 | 0.35 | - |  |  |  |  |  |  |  |  |  |
| El Porvenir (Córdoba) | 0.06 | 0.00 | 0.00 | 0.04 | 0.08 | - |  |  |  |  |  |  |  |  |
| Ibagué (Tolima) | 4.02 | 0.03 | 2.15 | 0.15 | 0.00 | 0.06 | - |  |  |  |  |  |  |  |
| Medina (Cundinamarca) | 0.04 | 0.00 | 0.00 | 0.03 | 0.06 | 0.00 | 0.06 | - |  |  |  |  |  |  |
| Nuevo Leon | 0.02 | 0.03 | 0.03 | 0.02 | 0.03 | 0.03 | 0.03 | 0.03 | - |  |  |  |  |  |
| Piedras (Tolima) | 0.12 | 0.01 | 0.02 | 0.05 | 0.06 | 0.03 | 0.07 | 0.02 | 0.01 | - |  |  |  |  |
| Pipiral (Meta) | 0.07 | 0.14 | 0.00 | 0.06 | 0.44 | 0.00 | 0.13 | 0.00 | 0.06 | 0.04 | - |  |  |  |
| Puente Quetame (Cundinamarca) | 0.08 | 0.05 | 0.06 | 0.05 | 0.31 | 0.02 | 0.15 | 0.25 | 0.02 | 0.03 | 0.31 | - |  |  |
| Puerto Gaitán (Meta) | 0.04 | 0.00 | 0.00 | 0.03 | 0.04 | 0.00 | 0.04 | 0.00 | 0.02 | 0.02 | 0.00 | 0.03 | - |  |
| San Martín (Meta) | 0.00 | 0.00 | 0.00 | 1.16 | 0.00 | 0.00 | 0.00 | 0.00 | 0.04 | 0.06 | 0.00 | 0.00 | 0.00 | - |
| Tamaulipas | 0.01 | 0.01 | 0.01 | 0.01 | 0.01 | 0.01 | 0.02 | 0.01 | 0.00 | 0.01 | 0.02 | 0.01 | 0.01 | 0.01 |
