# Supplementary material for: A preliminary assessment of population genetic structure of the common vampire bat (Desmodus rotundus) in Colombia
Source: PeerJ. 2025 Nov 10;13:e20306. doi: 10.7717/peerj.20306 (PMC12614099; doi:10.7717/peerj.20306)
Supplement: Supplemental Information 7 — Migrant identification was conducted in GeneClass 2.0 (Piry et al., 2004) . This software identifies likely first-generation migrants (shown in red) and the likelihood that these migrants originated from other sampled populations (Piry et al., 2004) . The lowest metrics of -log(L) indicate the most likely population of origin (shown in green). 15 first-generation migrants were identified. [file peerj-13-20306-s007.docx]

**Table S4: First-generation migration probability between populations.** Migrant identification was conducted in GeneClass 2.0 (Piry et al., 2004). This software identifies likely first-generation migrants (shown in red) and the likelihood that these migrants originated from other sampled populations (Piry et al., 2004). The lowest metrics of -log(*L*) indicate the most likely population of origin (shown in green). 15 first-generation migrants were identified.

| ***D. rotundus* Sample** | **Population of Collection** | **-log (*L*_home / *L*_max)** | **Probability** | **-log(*L*) Pop1** | **-log(*L*) Pop2** | **-log(*L*) Pop3** |
| --- | --- | --- | --- | --- | --- | --- |
| W6_100303_1 | 1 | 0.000 | 0.50 | 10.869 | 28.749 | 28.344 |
| W6_100303_144 | 1 | 0.000 | 0.5000 | 9.766 | 23.146 | 22.740 |
| W6_100303_22 | 1 | 0.000 | 0.5000 | 13.310 | 35.329 | 34.737 |
| W6_100303_61 | 1 | 0.000 | 0.5000 | 11.035 | 28.865 | 28.147 |
| W6_100303_82 | 1 | 0.000 | 0.5000 | 14.541 | 27.551 | 27.994 |
| W6_100303_94 | 1 | 0.000 | 0.5000 | 16.306 | 28.759 | 27.445 |
| 1_110 | 2 | 1.019 | 0.0573 | 19.061 | 7.362 | 6.343 |
| 108148_133 | 2 | 0.000 | 0.6674 | 25.699 | 9.002 | 9.217 |
| 118166_140 | 2 | 0.000 | 0.6550 | 36.079 | 13.820 | 14.378 |
| 119167_134 | 2 | 0.000 | 0.6538 | 33.334 | 15.159 | 15.476 |
| 120168_136 | 2 | 0.839 | 0.1232 | 34.113 | 15.163 | 14.324 |
| 186226_11 | 2 | 1.086 | 0.0941 | 29.299 | 12.460 | 11.374 |
| 187227_81 | 2 | 0.360 | 0.2318 | 34.539 | 14.760 | 14.401 |
| 2_128 | 2 | 3.048 | 0.0031 | 32.743 | 16.441 | 13.392 |
| 201241_39 | 2 | 0.000 | 0.6574 | 34.238 | 13.720 | 14.845 |
| 2034_112 | 2 | 0.000 | 0.6523 | 31.635 | 12.740 | 12.839 |
| 211251_103 | 2 | 0.000 | 0.6594 | 31.715 | 10.850 | 11.451 |
| 218258_82 | 2 | 0.891 | 0.1161 | 32.937 | 16.427 | 15.536 |
| 219259_97 | 2 | 0.000 | 0.6521 | 31.908 | 11.732 | 12.247 |
| 2337_138 | 2 | 1.459 | 0.0545 | 31.760 | 12.737 | 11.278 |
| 2784_55 | 2 | 0.618 | 0.1618 | 35.097 | 14.300 | 13.682 |
| 3_123 | 2 | 2.066 | 0.0204 | 34.289 | 14.109 | 12.044 |
| 3964_113 | 2 | 1.327 | 0.0684 | 35.222 | 13.084 | 11.757 |
| 4269_108 | 2 | 2.851 | 0.0048 | 30.380 | 13.947 | 11.096 |
| 4467_130 | 2 | 0.765 | 0.1439 | 33.812 | 15.052 | 14.287 |
| 4467_22 | 2 | 1.220 | 0.0787 | 29.253 | 13.266 | 12.045 |
| 5475_72 | 2 | 0.000 | 0.6590 | 32.651 | 11.905 | 13.636 |
| 5578_104 | 2 | 0.230 | 0.2531 | 33.362 | 15.115 | 14.885 |
| 5682_57 | 2 | 0.638 | 0.1632 | 29.444 | 14.284 | 13.647 |
| 6086_87 | 2 | 0.259 | 0.2512 | 28.146 | 10.580 | 10.321 |
| 6187_120 | 2 | 0.909 | 0.1132 | 33.766 | 13.492 | 12.583 |
| 6490_73 | 2 | 2.363 | 0.0141 | 26.141 | 14.772 | 12.408 |
| 6490_86 | 2 | 0.000 | 0.6570 | 25.266 | 10.469 | 11.193 |
| 6798_84 | 2 | 0.000 | 0.6567 | 31.192 | 9.818 | 9.911 |
| 9_137 | 2 | 0.403 | 0.2150 | 34.635 | 10.957 | 10.554 |
| H2_114 | 2 | 1.651 | 0.0441 | 36.732 | 14.291 | 12.640 |
| H33_116 | 2 | 0.000 | 0.6600 | 34.113 | 12.315 | 12.912 |
| H9_115 | 2 | 0.000 | 0.6568 | 36.033 | 15.004 | 15.323 |
| Pipi3_205 | 2 | 0.000 | 0.6506 | 30.919 | 17.244 | 18.888 |
| Pipi4_148 | 2 | 0.247 | 0.2248 | 35.523 | 19.241 | 18.994 |
| Pipi4_206 | 2 | 0.000 | 0.6538 | 36.079 | 19.780 | 20.692 |
| Queta1_198 | 2 | 0.112 | 0.2725 | 34.079 | 17.641 | 17.529 |
| Queta2_199 | 2 | 0.000 | 0.6527 | 33.556 | 12.351 | 13.369 |
| Queta3_200 | 2 | 0.672 | 0.1591 | 34.778 | 12.119 | 11.446 |
| Queta4_201 | 2 | 0.000 | 0.6541 | 33.125 | 17.473 | 19.273 |
| Queta5_202 | 2 | 0.000 | 0.6551 | 32.077 | 15.909 | 17.122 |
| Queta6_203 | 2 | 0.000 | 0.6524 | 32.952 | 15.666 | 17.312 |
| RZ2_208 | 2 | 0.000 | 0.6588 | 29.980 | 13.689 | 14.655 |
| 1065_3 | 3 | 1.035 | 0.0645 | 30.016 | 10.553 | 11.588 |
| 116164_101 | 3 | 0.596 | 0.1293 | 35.018 | 12.791 | 13.387 |
| 118166_135 | 3 | 0.000 | 0.6326 | 31.760 | 12.922 | 12.206 |
| 119164_119 | 3 | 1.975 | 0.0146 | 34.158 | 19.473 | 21.448 |
| 185225_109 | 3 | 0.456 | 0.1547 | 33.493 | 11.790 | 12.246 |
| 187227_63 | 3 | 0.000 | 0.6334 | 31.204 | 13.002 | 12.220 |
| 190230_62 | 3 | 0.066 | 0.2432 | 34.033 | 12.799 | 12.865 |
| 200240_40 | 3 | 0.225 | 0.2071 | 30.937 | 13.307 | 13.532 |
| 206246_34 | 3 | 2.592 | 0.0052 | 35.602 | 13.433 | 16.024 |
| 209249_121 | 3 | 0.920 | 0.0860 | 32.079 | 10.215 | 11.134 |
| 2135_98 | 3 | 0.529 | 0.1481 | 32.635 | 11.772 | 12.301 |
| 220260_65 | 3 | 0.000 | 0.6289 | 32.431 | 13.488 | 12.761 |
| 2381_17 | 3 | 0.000 | 0.6319 | 34.635 | 16.412 | 14.162 |
| 2541_4 | 3 | 0.000 | 0.6309 | 30.266 | 16.129 | 13.806 |
| 289229_49 | 3 | 0.595 | 0.1262 | 31.176 | 12.667 | 13.262 |
| 3553_68 | 3 | 0.000 | 0.6304 | 27.891 | 14.677 | 13.680 |
| 3656_131 | 3 | 0.000 | 0.6354 | 34.845 | 13.260 | 12.211 |
| 3656_71 | 3 | 0.000 | 0.6342 | 35.715 | 12.656 | 11.466 |
| 3758_105 | 3 | 0.000 | 0.6311 | 31.061 | 18.318 | 17.281 |
| 4_124 | 3 | 0.000 | 0.6324 | 31.192 | 12.325 | 11.967 |
| 4370_102 | 3 | 0.481 | 0.1489 | 29.459 | 11.460 | 11.942 |
| 4467_74 | 3 | 0.910 | 0.0874 | 33.556 | 13.122 | 14.032 |
| 4571_111 | 3 | 0.000 | 0.6338 | 29.459 | 16.534 | 13.835 |
| 4980_24 | 3 | 0.232 | 0.2089 | 28.901 | 14.020 | 14.251 |
| 5_126 | 3 | 0.000 | 0.6327 | 31.569 | 17.346 | 16.437 |
| 5373_106 | 3 | 0.926 | 0.0848 | 25.600 | 11.490 | 12.416 |
| 5475_132 | 3 | 1.421 | 0.0393 | 29.459 | 11.179 | 12.600 |
| 5885_67 | 3 | 0.000 | 0.6302 | 34.334 | 14.656 | 13.579 |
| 6_125 | 3 | 0.389 | 0.1746 | 34.095 | 12.209 | 12.597 |
| 6389_91 | 3 | 0.358 | 0.1877 | 37.431 | 12.700 | 13.058 |
| 6591_99 | 3 | 1.197 | 0.0600 | 33.715 | 14.134 | 15.331 |
| 6595_129 | 3 | 0.000 | 0.6351 | 31.378 | 10.967 | 10.863 |
| 6894_107 | 3 | 2.655 | 0.0026 | 36.635 | 16.990 | 19.645 |
| 7_139 | 3 | 0.491 | 0.1507 | 34.493 | 13.250 | 13.741 |
| 77125_78 | 3 | 1.281 | 0.0480 | 31.697 | 13.254 | 14.535 |
| 79_100 | 3 | 0.000 | 0.6334 | 31.414 | 12.526 | 12.460 |
| 8_127 | 3 | 0.000 | 0.6297 | 32.255 | 12.435 | 12.315 |
| 85133_122 | 3 | 2.141 | 0.0101 | 35.511 | 14.640 | 16.781 |
| H37_117 | 3 | 0.404 | 0.1609 | 29.186 | 14.142 | 14.546 |
| H58_118 | 3 | 0.000 | 0.6358 | 32.937 | 14.329 | 14.110 |
| Pipi1_146 | 3 | 1.186 | 0.0456 | 26.194 | 15.105 | 16.291 |
| Pipi3_147 | 3 | 1.443 | 0.0332 | 36.192 | 21.190 | 22.634 |
| Queta7_204 | 3 | 2.889 | 0.0015 | 31.386 | 16.719 | 19.608 |
| RZ1_207 | 3 | 1.412 | 0.0320 | 31.812 | 12.745 | 14.157 |
